# Semi-Supervised Clustering for Identification of MCI and Dementia Cohorts with a Brief Digital Cognitive Assessment

**DOI:** 10.21203/rs.3.rs-8532476/v1

**Published:** 2026-01-13

**Authors:** Daniel Schulman, Ali Jannati, Tanya Talkar, David J. Libon, Rod Swenson, Connor Higgins, Alvaro Pascual-Leone, Sean Tobyne

**Affiliations:** 1 Linus Health, Inc., Boston, MA, USA; 2 Harvard Medical School, Department of Neurology, Boston, MA, USA; 3 Rowan University, New Jersey Institute for Successful Aging, School of Osteopathic Medicine, Stratford, NJ, USA; 4 Rowan University, Department of Psychology, Glassboro, NJ, USA; 5 University of North Dakota, School of Medicine and Health Sciences, Grand Forks, ND, USA; 6 Hebrew SeniorLife, Deanna and Sidney Wolk Center for Memory Health and Hinda and Arthur Marcus Center for Aging Research, Boston, MA, USA

## Abstract

Traditional neuropsychological assessment for diagnosis of mild cognitive impairment (MCI) or dementia requires a lengthy in-clinic evaluation by a specialist. This creates a substantial patient burden and prolonged diagnostic and treatment timelines. Digital cognitive assessments (DCA) offer a scalable solution to meet these challenges, but their validation is challenged by the scarcity of large, high-quality datasets with established ground truth. We applied a semi-supervised model-based clustering method to combine a large dataset (N=1189) of the Digital Assessment of Cognition (DAC), a brief, remote-capable DCA, with a smaller dataset pairing DAC assessments with ground-truth neuropsychological diagnoses (N=248). The resulting model identified cognitively unimpaired, MCI, and dementia groups with high accuracy on an external test dataset. Congruent validity was established through strong expected associations with traditional analog assessments. These results validate prior exploratory work and demonstrate the potential for more nuanced, holistic, and scalable cognitive assessments in non-specialist settings.

## Introduction

Dementia is a major public health concern with profound implications for healthcare systems worldwide. Based on the Global Burden of Disease Study 2019, Nichols et al.^1^ project that the global prevalence of dementia would rise from 57.4 million in 2019 to 152.8 million by 2050, largely driven by population growth and demographic aging. Consequently, substantial advocacy and policy initiatives have emerged to promote earlier detection and diagnosis of dementia^2,3^ and mild cognitive impairment (MCI).^4^ Urgency for early detection is driven, in part, by strong evidence that addressing modifiable risk factors can reduce the risk of developing dementia,^5^ and that targeted cardiovascular risk and lifestyle interventions can improve cognitive function in individuals even when MCI is present.^6^

However, the availability of the necessary services to diagnose MCI and dementia remains poor, and is a barrier to providing available interventions. A recent systematic review and meta-analysis^7^ reported an average delay of 3.5 years from first reported symptoms to clinical diagnosis, which extended to an average of 4.1 years in early-onset dementia. Considering that 8–14% of patients with MCI progress to dementia each year^8-10^, a delay of several years means that by the time a diagnosis is made, and available treatment strategies are considered, the disease has often progressed to a more advanced stage where treatments may be less effective^11-13^.

Furthermore, underdiagnosis of cognitive impairment presents a significant, unrecognized burden on health systems. A recent large-scale European survey reported underdiagnosis rates of 43–87% for dementia across 19 countries.^14^ Similarly, a study based on the US Health and Retirement Survey estimated that 42% of cases of dementia are undiagnosed, with significant racial and ethnic disparities.^15^ Underdiagnosis of MCI is even more common; a study of the US Medicare population estimated that more than 90% of MCI cases remain undiagnosed^16^.

Traditional paper and pencil neuropsychological testing is time-consuming and burdensome for patients. Moreover, specialist availability remains inadequate, contributing to prolonged delays in diagnosis and persistent underdiagnosis^17^. Brief digital cognitive assessments have been advocated as a promising approach to overcome these deficiencies.^18^ Recent advances have generated substantial research interest in remote and/or unsupervised digital assessments, which enable diverse clinical workflows^19^. The optimal approach requires that a brief digital cognitive assessment be developed and validated against a large dataset of gold-standard diagnoses based on traditional neuropsychological tests. Such validation remains uncommon, as most prior studies rely on either small datasets or validate against older brief assessments, which themselves may lack sufficient accuracy or validity.^20^ Developing and validating new assessments against existing traditional assessments risks perpetuating their inherent limitations, while validating against small gold-standard datasets may compromise generalizability, particularly as computational models increase in complexity. Berisha et al. elucidate this issue in the context of the “curse of dimensionality”, a well-established concept in statistical learning theory, providing an example of the impact on real-world performance of cognitive assessments.^21^

The inherent challenge lies in obtaining large gold-standard datasets, given the substantial patient burden of comprehensive neuropsychological evaluation and the limited availability of datasets with ground-truth diagnosis. In this work, we explore an approach based on semi-supervised learning: a family of statistical and machine-learning methods that combine supervised learning on a small dataset of labeled data with unsupervised learning on a larger dataset of unlabeled data.^22^ Here, labeled data comprises digital cognitive assessment outputs paired with gold-standard clinical diagnoses from comprehensive neuropsychological evaluation, while unlabeled data consists of digital cognitive assessment outputs alone.

The Linus Health Digital Assessment of Cognition (DAC) is a brief DCA that incorporates digital adaptations of several traditional neuropsychological tests^23^. The DAC can be completed in as little as 7 to 8 minutes and can be administered remotely without specialized hardware, via internet-connected consumer smartphone, laptop, desktop, or tablet devices. DAC incorporates the 6-word Philadelphia (repeatable) Verbal Learning Test (PrVLT), animal fluency test, and the Backward Digit Span Test (BDST)^23^. This allows for an efficient and accessible means of assessing key cognitive domains relevant to emerging cognitive impairment, including verbal learning and memory, semantic memory and lexical access, executive function, and processing speed. In prior exploratory work, k-means clustering methods were applied to metrics output by the DAC, yielding clusters that were interpretable as cognitively unimpaired (CU), amnestic MCI, dysexecutive MCI, and probable dementia. This work found a high degree (>90%) of concordance between DAC-based clusters and clinical diagnosis based on comprehensive neuropsychological evaluation^23^.

The primary goal of this study was to develop a model capable of differentiating between CU individuals, those with MCI, and those with probable dementia using exclusively DAC-derived metrics. To ensure clinical utility, we required that the model demonstrate robust discriminatory power against gold-standard diagnoses (as opposed to relying on screening assessments like the Mini-Mental Status Examination (MMSE), Montreal Cognitive Assessment (MoCA), or Saint Louis University Mental Status (SLUMS) examination), output well-calibrated probabilities, and generate clinically interpretable results with strong face validity.

The key methodological challenge was the limited availability of large, high-quality datasets pairing a digital assessment with gold-standard clinical diagnoses. We addressed this challenge through a novel semi-supervised model-based clustering method that integrates a comparatively small dataset pairing DAC metrics that includes clinical diagnosis with a larger dataset of DAC metrics alone.

As contributions, we describe a novel methodology that uses semi-supervised clustering methods to generate a high-quality, clinically applicable model for identifying both MCI and probable dementia while maintaining feasible data requirements. Applying this methodology, we present a state-of-the-art model for cognitive classification based only on a brief, automated, remote-capable digital cognitive assessment. Finally, we demonstrate multiple aspects of the model’s validity: face validity (clinically interpretable clusters), congruent validity (expected associations with demographics and other assessments), and predictive validity (accurate diagnosis prediction on a held-out test set).

## Results

### Datasets

The evaluable dataset of DAC results from the Apheleia-001 study consisted of N=1189 participants. This dataset is unlabeled and does not include a clinical diagnosis of mild cognitive impairment and/or dementia. The Rowan dataset included N=248 individuals,^24,25^ split randomly into training (N=175) and held-out testing (N=75) sets. Table 1 summarizes participant demographics and scores on cognitive assessments, including the MMSE and the Linus Health Digital Clock and Recall (DCR).

### Measurements

The DAC includes a 6-word version of the Philadelphia Verbal Learning Test (PrVLT)^26^, a 60-second animal fluency test^27^, and 3 trials of a 5-digit Backward Digit Span Test (BDST). The P(r)VLT includes two immediate free recall test trials, a delayed free recall task, followed by a delayed recognition task in which the individual is given a forced choice between an original target word, a prototypical foil, and a generic foil from the same semantic category. The semantic fluency and BDST are presented during the interval between the second trial of the immediate-recall task and the delayed free recall task.

We selected *a priori*, via literature review and expert consensus, a small set of measures with strong evidence of association with amnestic and/or dysexecutive cognitive impairment:

- BDST: Total digits correctly recalled in serial order, across three trials (range: 0-15)^28,29^
- Animal naming: Number of total unique animals^30^
- P(r)VLT delayed recall: Words correctly recalled (range: 0-6)^26,31^
- P(r)VLT delayed recognition: Correct words, prototypical foils, and generic foils selected (range: 0-6, totaling 6)^26,31^

The DAC, as administered in the Apheleia-001 study, also included a brief screening for depression, anxiety, and apathy. Depression and anxiety were assessed by the 4-item version of the Patient Health Questionnaire for Depression and Anxiety (PHQ-4)^32^. Apathy was assessed by two items adapted from the Neuropsychiatric Inventory Questionnaire (NPI-Q)^33^. As these items are not intended to measure cognitive impairment, they were not included in the clustering model. However, they were used subsequently in the assessment of congruent validity.

### Clustering Model Training

We iteratively developed a semi-supervised finite mixture model (FMM), based on examination of model fit, residual diagnostics, and face validity of the resulting clusters. While the Rowan data were labeled with 3 diagnostic classes (CU, MCI, Dem), we did consider models that allowed multiple clusters per label once combined with the Apheleia dataset. This process produced a 6-cluster model, with 3 MCI clusters and 2 probable dementia clusters (Table 2). Descriptions of the MCI and dementia clusters were produced by informal discussion and consensus, following examination of the model. Note that the subtype clusters are combined when MCI and Dem are reported without modifiers (see Table 2).

A separate probability model was selected for each metric or group of metrics. (Table 3). Metrics were modeled independently, without accounting for correlation between them. We applied “Conway-Maxwell” generalizations of common probability distribution families^34^, which allow for the variance of the metrics to differ between clusters.

In some cases, the probability models were modified to improve model fit and/or diagnostics, and to improve the interpretability of the resulting clusters. For the total number of unique animals in the animal naming task, we selected a zero-inflated Conway-Maxwell Poisson distribution^35^ to account for excess zero scores. Similarly, to account for excess zeros in the number of words correct in the PVLT-6 delayed free recall task, we selected a zero-inflated Conway-Maxwell Binomial distribution. To account for a greater number of perfect scores (15) than expected in the BDST, we selected an N-inflated^36^ Conway-Maxwell Binomial distribution.

### Cluster Data Distributions

**Figure 1.**
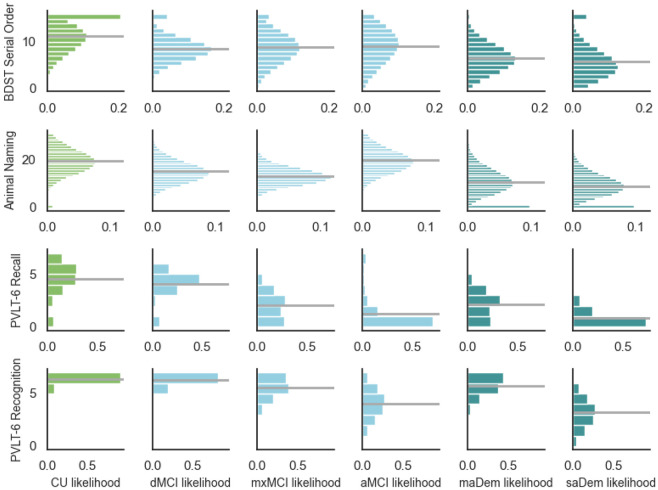
Probability mass functions (PMFs) of the clusters in the fitted models. The horizontal grey lines indicate cluster means. For simplicity, the PMF for the PVLT-6 recognition task shows only the distribution of the number of correct responses chosen, and not the foils chosen.

Table 4 gives estimated cluster means and standard deviations for each metric. Figure 1 illustrates the full probability mass functions (PMFs).

### Congruent Validity

We examined several variables in the Apheleia dataset that were not part of the clustering model, including demographics, other cognitive assessments, and screenings for affective disorders. Based on prior literature, we hypothesized that:

- Age would be higher in clusters with greater impairment.^39^
- Education would be lower in clusters with greater impairment.^39^
- MMSE score would be lower in clusters with greater impairment.^40^
- The DCTclock Score^41^ from the Linus Health DCR would be lower in clusters with greater impairment.^42^
- The delayed recall score from the Linus Health DCR would be lower in clusters with greater impairment.^42^
- Screening assessments of affective disorders (depression, anxiety, and apathy) would be higher in impaired clusters compared to the unimpaired cluster. We did not hypothesize differences between mild cognitive impairment and probable dementia clusters, as the prior literature does not show a consistent relationship between affective disorders and the stage or severity of dementia^43^.

To test these hypotheses, we collapsed model outputs to three clusters (cognitively unimpaired, mild cognitive impairment, and probable dementia). Since all variables we examined were not normally distributed, we relied on nonparametric statistical tests. We conducted a series of generalized Kruskal-Wallis tests^44^ for between-cluster differences. Significant results were followed by post hoc pairwise comparisons, using a corresponding generalization of Dunn’s test.

In all cases, the differences between clusters were highly significant, and pairwise differences were significant and in the hypothesized direction (Table 5). For demographic variables (age, education) and assessments of cognitive impairment (MMSE, DCR), all differences were highly significant (p<0.001). The evidence of between-cluster differences for screening assessments of affective disorders (depression, anxiety, apathy) was weaker; nevertheless, we found significant increases in reports of affective disorder symptoms between the CU cluster and impaired clusters in all cases. Although not hypothesized, we found significantly greater reports of depression and anxiety in probable dementia clusters compared to MCI clusters.

### Exploration of MCI and Probable Dementia Clusters

To better understand the characteristics of the MCI and probable dementia clusters, we performed a similar analysis of the same set of variables on the Apheleia datasets, comparing all 6 clusters. The statistical methods are unchanged; however, as this is exploratory we did not give hypotheses.

#### Age

**Figure 2.**
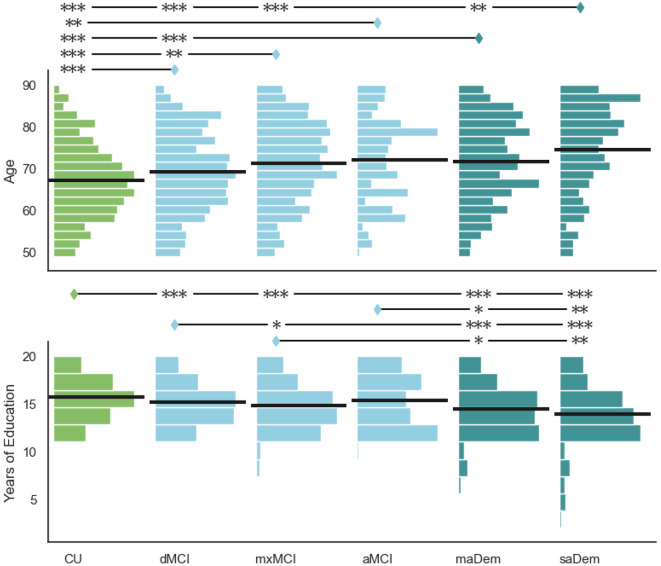
Between-cluster comparison of demographic variables. Horizontal lines within histograms indicate means. Clusters annotated with diamonds have significantly higher values than clusters annotated with stars on the same line: * p ≤ 0.05; ** p ≤ 0.01; *** p ≤ 0.001.

There was a significant between-cluster difference in years of age (H=86.01, p<0.001), as illustrated in the upper row of Figure 2. Examining pairwise differences, generally we found that individuals in clusters indicative of greater cognitive impairment overall, and greater amnestic impairment specifically, were older. Individuals in all impaired clusters were significantly older than individuals in the CU cluster.

Individuals in the mxMCI cluster were significantly older than individuals in the dMCI cluster (Z=3.77, p=0.001). Individuals in the aMCI cluster were near-significantly older than individuals in the dMCI cluster (Z=2.21, p=0.083). Individuals in both probable dementia clusters were significantly older than individuals in the dMCI cluster, and individuals in the saDem cluster were significantly older than individuals in the mxMCI cluster (Z=4.13, p<0.001). Individuals in the saDem cluster were significantly older than individuals in the maDem cluster (Z=3.56, p=0.002).

#### Education

There was a significant between-cluster difference in reported years of education (H=73.26, p<0.001), as illustrated in the lower row of Figure 2. Generally, we found that individuals in clusters indicative of greater cognitive impairment reported fewer years of education. With the exception of the aMCI cluster, individuals in all impaired clusters reported significantly fewer years of education than individuals in the CU cluster. Individuals in the mxMCI cluster reported significantly fewer years of education than individuals in the dMCI cluster (Z=-2.61, p=0.035). Individuals in probable dementia clusters reported significantly fewer years of education than individuals in MCI clusters. There was also no significant difference in education between the two probable dementia clusters.

#### MMSE

**Figure 3.**
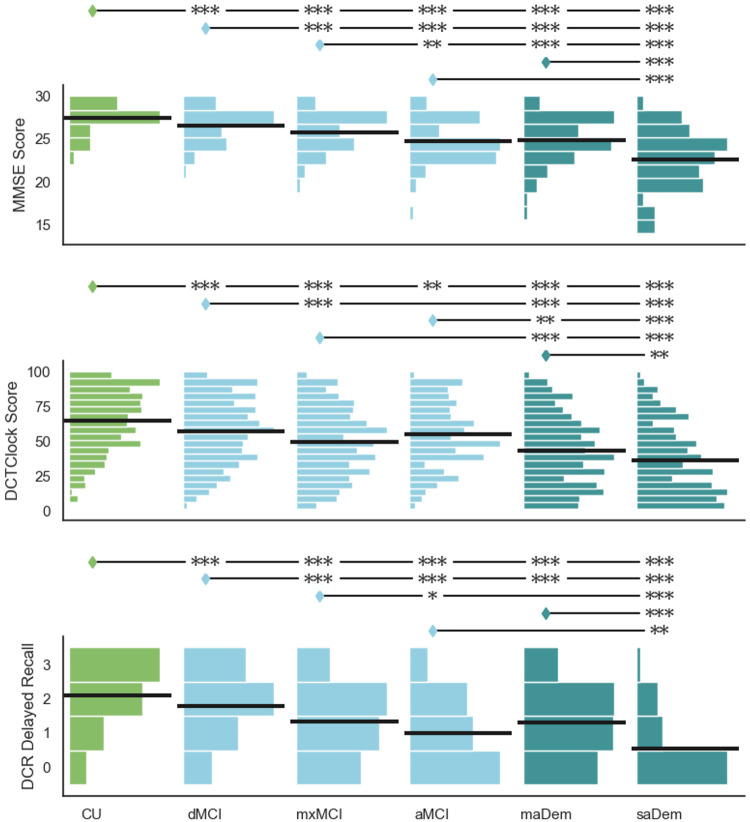
Between-cluster comparison of cognitive assessments. Horizontal lines within histograms indicate means. Clusters annotated with diamonds have significantly higher values than clusters annotated with stars on the same line: * p ≤ 0.05; ** p ≤ 0.01; *** p ≤ 0.001.

There was a significant difference in MMSE scores between CU, MCI, and probable dementia clusters (H=393.88, p<0.001), as illustrated in the upper row of Figure 3. Individuals in more impaired clusters had lower MMSE scores. There were significant differences between all three MCI clusters, with individuals in clusters consistent with greater amnestic impairment receiving lower MMSE scores. Individuals in the aMCI cluster had significantly lower MMSE scores than individuals in the mxMCI cluster (Z=-3.28, p=0.001), or individuals in the dMCI cluster (Z=-5.49, p<0.001). Individuals in the mxMCI cluster had significantly lower MMSE scores than individuals in the dMCI cluster (Z=−5.35, p<0.001). Similarly, individuals in the saDem cluster had significantly lower MMSE scores than individuals in the maDem cluster (Z=−7.29, p<0.001).

#### DCR Clock Drawing

There was a significant difference in DCTclock Scores between CU, MCI, and probable dementia clusters (H=229.39, p<0.001), as illustrated in the middle row of Figure 3. Individuals in more impaired clusters had lower DCTclock scores. Individuals in the mxMCI cluster had significantly lower scores than individuals in the dMCI cluster (Z=−5.45, p<0.001). We observed a trend toward lower scores compared to individuals in the aMCI cluster (Z=−1.86, p=0.088). Individuals in the saDem cluster had significantly lower scores than individuals in the maDem cluster (Z=−3.21, p=0.004).

#### DCR Delayed Recall

There was a significant difference in the number of words correctly recalled in the DCR delayed recall task between CU, MCI, and probable dementia clusters (H=309.40, p<0.001), as illustrated in the lower row of Figure 3. Individuals in more impaired clusters recalled fewer words. There were significant differences between all three MCI clusters, with individuals in clusters consistent with greater amnestic impairment recalling fewer words. Individuals in the aMCI cluster recalled significantly fewer words than individuals in the mxMCI cluster (Z=−2.58, p=0.022), or individuals in the dMCI cluster (Z=−5.78, p<0.001). Individuals in the mxMCI cluster recalled significantly fewer words than individuals in the dMCI cluster (Z=−7.48, p<0.001). Similarly, individuals in the saDem cluster recalled significantly fewer words than individuals in the maDem cluster (Z=−8.41, p<0.001).

#### Depression

**Figure 4.**
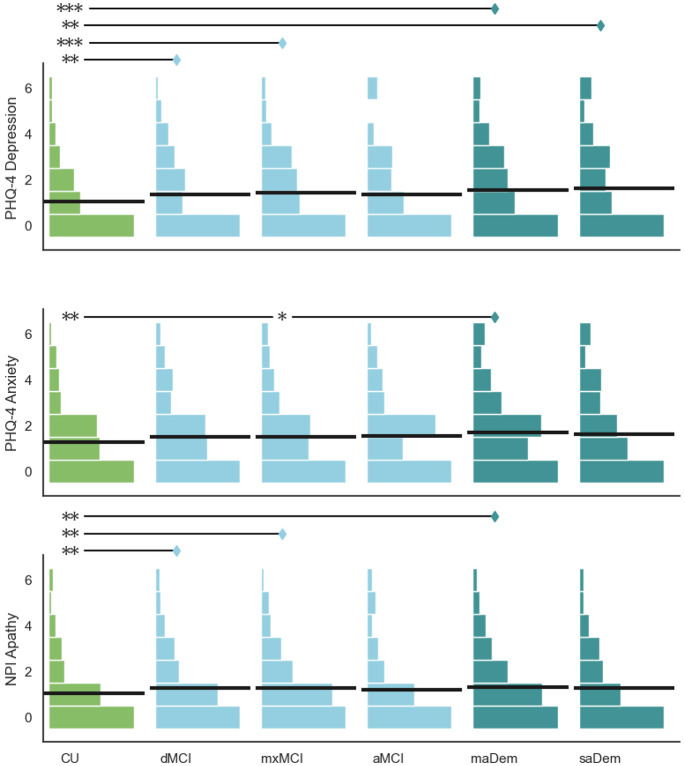
Between-cluster comparison of affective assessments. Horizontal lines within histograms indicate means. Clusters annotated with diamonds have significantly higher values than clusters annotated with stars on the same line: * p ≤ 0.05; ** p ≤ 0.01; *** p ≤ 0.001.

There was a significant difference between clusters in the scores on the PHQ-4 depression screening (H=27.90, p<0.001), as illustrated by the upper row of Figure 4. With the exception of the aMCI cluster, all impaired clusters had significantly higher scores than the CU cluster (dMCI: Z=3.52, p=0.004; mxMCI: Z=4.11, p<0.001; maDem: Z=4.88, p<0.001; saDem: Z=3.66, p=0.003). There were no significant differences between any MCI or probable dementia clusters.

#### Anxiety

There was a significant difference between clusters in the scores on the PHQ-4 anxiety screening (H=18.73, p=0.002), as illustrated by the middle row of Figure 4. Individuals in the maDem cluster had significantly higher scores than those in the CU cluster (Z=3.86, p=0.001) or the mxMCI cluster (Z=2.98, p=0.028). There were no significant pairwise differences between any other clusters.

#### Apathy

There was a significant difference between clusters in the scores on the apathy assessment (H=17.73, p=0.003), as illustrated by the lower row of Figure 4. Individuals in the dMCI cluster had significantly higher scores than individuals in the CU cluster (Z=3.42, p=0.006), as did individuals in the mxMCI cluster (Z=3.40, p=0.006) and the maDem cluster (Z=3.56, p=0.004). There were no significant pairwise differences between any MCI or probable dementia clusters.

### Predictive Validity

To examine the model’s predictive validity, we compared the model’s cluster assignments to the gold-standard diagnosis on the held-out subset (N=75) of the Rowan dataset. As we are primarily interested in distinguishing between cognitively unimpaired, mild cognitive impairment, and dementia, the model’s output is collapsed to a 3-class classification. This 3-class classification is also more likely to be clinically informative as current diagnostic codes emphasize cognitive and functional impairment severity over cognitive impairment phenotypes. The overall performance of the DAC clustering model is summarized in the confusion matrix in Table 5. Overall accuracy was 78.7% (95% confidence interval [67.7%, 87.3%]).

**Figure 5.**
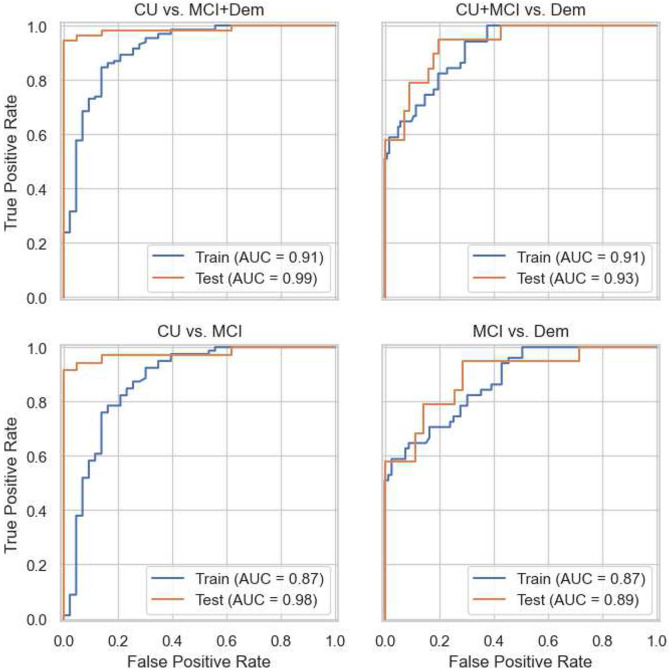
Receiver operating characteristic (ROC) curves showing performance of the model on classification tasks on the training and held-out test subsets of the Rowan dataset.

Table 6 summarizes performance on four clinically relevant binary classification tasks: Impairment detection (CU vs. MCI+Dem), dementia detection (CU+MCI vs. Dem), differentiating CU and MCI (assuming an individual does not have dementia), and differentiating MCI and dementia (assuming an individual is impaired). Sensitivity was higher on tasks that detected MCI (CU vs. MCI+Dem, CU vs. MCI), while specificity was good to excellent on all tasks. Positive predictive value (PPV) was also higher on tasks that detected MCI, while negative predictive value (NPV) was higher on tasks that detected probable dementia (CU+MCI vs. Dem, MCI vs. Dem). Discriminative ability (AUROC) was excellent on all tasks. Figure 5 displays ROC curves. Overall, the model’s performance was better on tasks that required discriminating CU from MCI than on tasks that discriminated between MCI and dementia.

To contextualize these results, we compared them to two simple baseline models, both of which use only age as a predictor: a 3-cluster model (with CU, MCI, and Dem clusters), and a 6-cluster model with the same structure as our final model (3 MCI clusters, and 2 probable dementia clusters). Both age-only models use a lognormal distribution to model patient age and are trained with the same semi-supervised methods as our final model. The model outperforms the baseline age-only models significantly (Table 7).

## Discussion

In this study, we developed and validated a probabilistic clustering model that accurately differentiates among cognitively unimpaired (CU) individuals, those with mild cognitive impairment (MCI), and those with probable dementia. The model achieved an overall strong discriminative performance on clinically relevant tasks of dissociating CU, MCI, and dementia when compared against an independent test dataset of gold-standard paper and pencil neuropsychological tests. The model is based exclusively on a small set of interpretable scores from the Linus Health Digital Assessment of Cognition (DAC), a brief, automated, and remote-capable digital cognitive assessment. We demonstrated the model’s robust congruent validity through expected associations with key demographic variables and independent cognitive assessments, and we rigorously evaluated its predicted performance on unseen data.

The central methodological contribution of this work is the use of a novel semi-supervised approach that combines a small labeled dataset with a much larger unlabeled dataset. This approach directly addresses a critical bottleneck in the development of ML-enabled models for cognitive classification: the scarcity of large, high-quality, gold-standard labeled datasets. We propose that this methodology is highly valuable and generalizable for validating such ML models in data-constrained environments.

While a few studies have applied semi-supervised learning to cognitive data, they have often relied on labels derived from traditional pencil-and-paper screening assessments (e.g., MMSE or MoCA scores) or required extensive neuropsychological batteries that limit their scalability and applicability for real-world clinical workflows. Yao et al.^45^ applied a pseudo-label with putback method to train a model that predicted cognitive impairment risk measured by the MMSE from features derived from medical examination data and the AD8 scale.^46,47^ This approach differs from ours in utilizing the MMSE as a criterion and employing an extensive feature set. A growing body of evidence shows that the MMSE and similar screening instruments should not be considered as gold standards in place of a full neuropsychological workup. Wang et al.^48^ demonstrated a novel semi-supervised method applied to deep learning models using the Alzheimer’s Disease Neuroimaging Initiative (ADNI) dataset.^49^ However, their approach required extensive neuropsychiatric test batteries that are not feasible in clinical practice.

### Performance in the Context of Existing Digital Assessments

A key contribution of our work is the model's high performance, particularly for detecting MCI. To contextualize these findings, we compared our results to published benchmarks for other digital cognitive assessments. For the crucial task of differentiating CU from MCI, our model achieved an AUROC of 0.977 on unseen data, with a sensitivity of 82.9% and perfect specificity. This compares favorably to other validated tools. For instance, digital adaptations of the Montreal Cognitive Assessment (MoCA) including MoCA Solo, MoCA Expresso, and MoCA Duo show accuracy ranging from 63–90% depending on population characteristics and cutoff thresholds ^50-52^. Meta-analytic data suggests digital MoCA variants achieve an overall AUROC of 0.84 for detecting amnestic mild cognitive impairment, though specificity varies considerably (27–95%) based on selected cutoffs^53^. The Computer Assessment of Mild Cognitive Impairment (CAMCI) reported a sensitivity of 86% and specificity of 94% for MCI detection^54^. While our sensitivity is comparable, our model's perfect specificity for correctly identifying cognitively unimpaired individuals is a notable strength, suggesting a very low false-positive rate for impairment. For broader dementia detection, our AUROC of 0.932 is also highly competitive. For example, a study of the BrainCheck assessment for staging dementia reported AUROCs ranging from 0.733 for mild vs. moderate dementia to 0.917 for mild vs. severe dementia^55^, whereas the digital Neurotrack Cognitive Assessment Battery (N-CAB) had up to 95% sensitivity and 89% specificity in distinguishing probable AD dementia from healthy aging ^56^. Likewise, the BioCog self-test reached 88–92% sensitivity and 82–87% specificity for detecting cognitive impairment in primary care, with an AUROC of 0.93 ^57^. A digital version of the Oxford Memory Task achieved an AUROC of 0.92 for differentiating subjective cognitive impairment from MCI ^58^. Therefore, our results position the DAC as a leading tool in the field, particularly for its high specificity in identifying impairment.

### Clinical Implications and Phenotypic Subtyping

Beyond overall cognitive classification, a key strength of our model is its ability to identify distinct, clinically meaningful subtypes of cognitive impairment. Our exploratory results indicate that the 6-cluster model differentiates between phenotypes characterized by predominantly amnestic versus dysexecutive deficits. For example, the aMCI cluster showed significantly lower delayed recall performance than the dMCI cluster, consistent with known amnestic vs non-amnestic MCI subtypes^59^. This suggests the 7-minute DAC can produce a nuanced cognitive profile that may previously have required a much longer assessment battery. This capability moves the assessment beyond its application as a cognitive impairment staging tool toward one capable of "phenotyping" the pattern of cognitive impairment an individual presents, which could have significant clinical utility for stratifying patients for clinical trials, predicting specific trajectories of cognitive decline, or tailoring non-pharmacological interventions. Future work should aim to validate these subtypes prospectively and determine their prognostic value.

### Limitations

Some limitations of this study should be acknowledged. First, there is a potential for spectrum bias. While our smaller labeled dataset (Rowan) was derived from a clinical setting, our larger unlabeled dataset (Apheleia) came from a clinical trial screening context. The Apheleia population, consisting of individuals with subjective memory complaints, may not fully represent the heterogeneity of the general healthcare population in which such a tool would be deployed.

A second, more subtle limitation arises from the semi-supervised methodology itself. With the unlabeled Apheleia dataset being substantially larger than the labeled Rowan training set, the fundamental structure of the six clusters is primarily driven by the characteristics of the Apheleia population. The model is therefore optimized to find patterns within this specific, self-selected group. Its generalizability to the more diverse spectrum of cognitive health and impairment seen in a general primary care setting remains to be established. True external validation on a prospectively recruited, unselected primary care cohort is thus the essential next step to confirm the model's real-world utility.

### Future Directions and Conclusion

These results were achieved using a small number of traditional, score-based metrics derived from the DAC. However, as a fully automated digital test, the DAC also captures a wealth of high-dimensional “process metrics”^60^ that are infeasible to collect in a manually scored test. Future work will incorporate these rich data streams. Examples include not only acoustic and speech-related features (e.g., response latencies, pause duration, vocal jitter and shimmer, spectral characteristics) that have been linked to cognitive load, but also behavioral metrics from the digital interface, such as response latencies in the recognition task^61,62^. Prior work has shown that including these process metrics can substantially improve the performance of other digital cognitive assessments, such as the clock-drawing tests^42^ and the trail-making tests^63^. Integrating these features has the potential to further improve the model's accuracy and its ability to detect the subtlest signs of cognitive change.

In conclusion, this study demonstrates that a brief, remote-capable digital cognitive assessment, when paired with a sophisticated semi-supervised learning model, can accurately and robustly identify individuals with MCI and dementia. The model's performance is competitive with or exceeds that of other digital assessments, and its ability to identify distinct clinical phenotypes offers a path toward more nuanced cognitive evaluation at scale. Applied in practice, this model could enable the DAC to serve as a key component in emerging clinical workflows in primary care and other non-specialist settings, thereby helping to close the profound gap in the early detection of cognitive impairment.^18,64^

## Methods

### Datasets

#### Unlabeled Dataset

The unlabeled dataset (Apheleia; N=1189) was assembled from participants in the Apheleia-001 study (ClinicalTrials.gov ID: NCT05364307; registration date 2022-05-06), organized by the Global Alzheimer's Platform Foundation (GAP) in collaboration with AbbVie, Inc. This trial was designed to investigate screening procedures, including the use of blood-based biomarkers, to reduce the screen failure rate among participants interested in therapeutic AD clinical trials. The goal was to identify and characterize participants with reported memory complaints and/or cognitive impairment using demographic information, clinical history, brief cognitive assessments, and blood-based biomarkers to enhance the probability of randomization^65^. Participants were recruited at 19 trial sites at locations in the continental United States and Canada. Eligible participants were between 50-90 years of age (inclusive) and had progressive cognitive complaints reported by the participant or caregiver. Participants who had reported or had a known negative amyloid PET scan in the past 24 months were excluded. Participants with a history of stroke within 6 months of prescreening and those with a diagnosis of a neurological disorder other than AD, that may be contributing to cognitive impairment, were excluded at the discretion of the investigators.

Participants completed a battery of paper and digital cognitive tests, demographic and medical information, and a blood draw for assessment of blood-based biomarkers for Alzheimer’s disease. Relevant to the work presented here, the demographic information included (among other variables) age and self-reported years of education. The cognitive tests for which data is available for this work included the Linus Health Digital Assessment of Cognition (DAC), the Linus Health Digital Clock and Recall (DCR), and the Mini-Mental State Examination (MMSE). The Apheleia-001 study was performed in accordance with the Declaration of Helsinki and its later amendments. The study procedures were explained to participants verbally and through written informed consent that was approved by the local IRB of each site participating in the GAP consortium (see the Apheleia study website (https://globalalzplatform.org/apheleia/) for a list of study sites). If, in the opinion of the site principal investigator, the participant did not have the capacity to sign the informed consent form, a legally authorized representative was used to grant consent on behalf of the participant.

#### Labeled Dataset

The labeled dataset (Rowan; N=248) was assembled from individuals recruited at three sources at Rowan-Virtua Health: the New Jersey Institute for Successful Aging Memory Assessment Program (NJISA MAP), outpatient referrals for neuropsychological assessment for suspected dementia, and the Departments of Geriatrics and Family Medicine outpatient ambulatory care services. The NJISA MAP program provides a comprehensive outpatient evaluation and work-up for suspected alterations involving cognition and personality/behavior. MAP patients were scheduled for three outpatient visits involving the administration of a neuropsychological protocol, an evaluation by a board-certified geriatric psychiatrist, and an evaluation by a clinical worker. Participants referred by their primary care provider for outpatient neuropsychological assessment because of suspected cognitive impairment underwent the same neuropsychological evaluation as MAP patients. Participants from the Rowan Departments of Geriatrics and Family Medicine were not referred or assessed clinically and did not undergo outpatient neuropsychological assessment. These participants were recruited for ongoing research on the development of digital neuropsychological assessment technology. Participants were excluded from this study if English was not their first language or if there was any history of head injury, substance abuse, a major psychiatric disorder such as major depression, another neurologic illness such as epilepsy, or metabolic disorders such as B12, folate, or a thyroid deficiency. For MAP and outpatient participants referred for neuropsychological evaluation, a knowledgeable family member was available to provide information regarding functional status. A full list of assessments administered for establishing gold standard diagnosis are available in the Supplementary Information. Across the participants in the labeled data, three separate neuropsychologists/geriatricians provided their diagnosis. This study was approved by the Rowan University Institutional Review Board, and consent was obtained that was consistent with the Declaration of Helsinki.

Following the detailed diagnosis produced by the neuropsychologists, participants were classified as cognitively unimpaired (CU), mildly cognitively impaired (MCI), or probable dementia (Dem). Participants were randomly split into a training set (70%; N=173) and a held-out test set (30%; N=75). The split was stratified by the diagnosing neuropsychologist to limit bias in the event of between-clinician differences in diagnostic approach. A comparison of the training and testing sets (Table 8) showed no statistically significant differences on a variety of demographics, cognitive tests, or diagnoses. The training set was used as labeled data for model development and combined with Apheleia data. The test set was reserved for validation and was not used to train or tune the model in any way.

### The Digital Assessment of Cognition

#### Assessment and Data Capture

**Figure 6.**
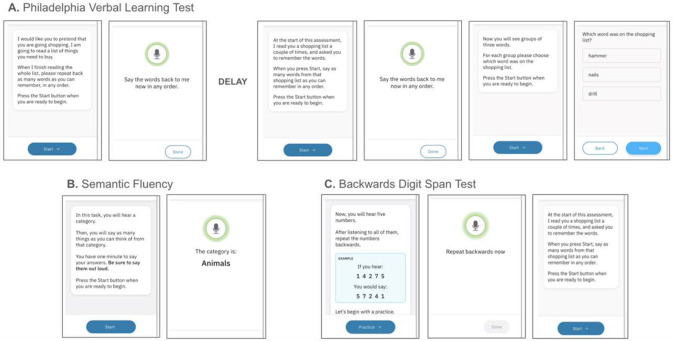
Overview of the DAC assessment components. (A) Philadelphia Verbal Learning Test (PVLT) immediate free recall trials are separated from PVLT delayed free recall trials by a delay, during which (B) semantic fluency, © backwards digit span (BDST), and a depression and anxiety screener (not shown) are completed. Following BDST, PVLT delayed recall and delayed recognition trials are completed.

DAC has been extensively described previously^23^. Briefly, the DAC includes, in order: (1) two immediate free recall trials of a 6-word Philadelphia (repeatable) Verbal Learning Test (PrVLT)^26^; (2) a 60 second semantic fluency “animal naming” task^27^; (3) three trials of 5-digit Backward Digit Span Test (BDST); (4) a 6-item depression and anxiety screening; (5) a delayed free recall trial of the 6-word P(r)VLT word list presented earlier; (6) and six delayed recognition trials of the 6-word P(r)VLT, in which the individual is presented with a forced choice between the prompted word, a prototypical foil, and a generic foil of the same semantic category (see Figure 6). For both the Apheleia and Rowan datasets, the DAC was administered with a tablet device. A trained examiner proctored the test administration, but all instructions were automated and delivered verbally by the device. All free recall responses, BDST responses, and animal naming responses are spoken by the participant. The administration device records speech for subsequent processing and analysis. Delayed recognition responses were recorded as physical touch using the iPad touchscreen.

#### Metric Definitions and Scoring

Following prior work^23^, in this paper we focus on the animal naming task, the BDST, and the delayed recall and delayed recognition tasks of the P(r)VLT. DAC scoring and outcome measures are a summary of the participant’s performance on the P(r)VLT-6, Semantic Fluency (‘Animals’), and BDST, including a breakdown of where points were earned in each component of this assessment, as listed below:

##### P(r)VLT-6:

- Immediate Recall (range 0-6): Total words correctly recalled from immediate free recall, trial 1.
- Immediate Recall Repeat (range 0-6): Total words correctly recalled from immediate free recall, trial 2.
- Delayed Recall (range 0-6): Total words correctly recalled from the delayed free recall test trial
- Delayed Recognition (range 0-6, with a total of 6): Total correct hits from the delayed recognition trial, and total number of prototypical and generic foils chosen.

##### Animal Fluency:

- Total unique animals: The total number of correct and unique ‘animal’ exemplars in 60 seconds and in each 15-second time epoch.

##### BDST:

- Total correct ANY order recall: Total number of digits correctly recalled regardless of their serial position.
- Total correct SERIAL order recall: Total number of digits correctly recalled in their exact serial position.

All audio recordings were passed through the Amazon Transcribe service API, yielding a JSON-formatted transcription that provides confidences and start and end times for each transcribed word, along with the final transcription. To improve transcription performance, the transcription call included a custom vocabulary word set that was determined based on the expected words during each of the tasks in the DAC (e.g., the six word list presented for P(r)VLT-6). Scoring for all tasks is completed through analysis of the transcript to assess whether words were recalled correctly in P(r)VLT-6 and BDST tasks, or to count the number of animals listed during the semantic fluency task. Animals were counted in reference to a corpus of 716 animals, after converting transcribed words to singular (e.g., ducks vs. duck). Participants were not given credit for perseverations or repeated animals, even if pluralized. All DAC scoring was completed in this way, excluding the Delayed Recognition task, which was not conducted through a speech-based assessment. Manual scoring of the ASR transcriptions for each task yielded a 5.77% word error rate in P(r)VLT-6 scores (both Immediate Recall trials and Delayed Recall), an 8.74% error rate in BDST scores, and a 6.78% error rate in Semantic Fluency Scores. These lie in the range of previously published Amazon Transcribe word error rates (WER), which have been reported from 2.50% to 9.00%^66-68^.

### Clustering Model Development

#### Problem Statement

Our primary goal was to produce a probabilistic clustering model capable of classifying individuals as CU, MCI, or probable dementia, using the set of scores produced by the DAC as described above. A probabilistic model yields an output that can be interpreted as a probability of impairment, which is potentially more clinically useful than a binary output of the presence or absence of impairment.

Extensive prior research has examined subtypes of MCI and dementia, such as amnestic and dysexecutive, with different presentations and prognoses.^59^ Therefore, we anticipated that the DAC scores within MCI and dementia groups may be heterogeneous and indicative of different patterns of impairment, and we considered models with multiple MCI clusters and/or multiple dementia clusters. We do not assume that these clusters will correspond to previously characterized MCI/dementia subtypes.

We treated this as a semi-supervised learning problem. The larger Apheleia dataset was treated as unlabeled data using only the DAC scores. The smaller Rowan dataset was treated as labeled data using both the DAC scores and the clinical diagnosis. Some observations in the Rowan dataset had only partial labels. For models with more than one MCI and/or dementia cluster, a diagnosis of MCI or dementia indicates that an individual should be assigned to one of the appropriate subset of clusters but does not indicate which one.

#### Semi-Supervised Finite Mixture Models

We fit a series of finite mixture models by maximum likelihood estimation, using a variant of the standard expectation-maximization (EM) algorithm. The algorithm was augmented with a binary constraint matrix, which is applied during the E-step of the EM algorithm. Wherever there is a zero in the constraint matrix, the posterior probability of cluster membership for an observation is constrained to zero.

This approach accommodates unlabeled, labeled, and partially labeled data. For example, consider a 4-cluster model, in which one cluster is designated as “CU” (cognitively unimpaired), two clusters are designated as “MCI”, and one cluster is designated as Dementia.

- Unlabeled: Observations are unconstrained and may belong to any cluster.
- Labeled: Observations labeled CU and Dementia are constrained to belong to the CU and the Dementia cluster, respectively, and cannot belong to any other cluster.
- Partially labeled: Observations labeled MCI are constrained to belong to one of the two MCI clusters, and cannot belong to the CU or dementia cluster.

For simplicity, we assume no correlation between DAC metrics and specify independent distributions for each metric.

#### Model Selection and Refinement

Models were developed iteratively. We began with a simple 3-cluster model (CU, MCI, dementia), and with a simple choice of probability distributions:

- Binomial distributions for BDST serial order score and for PVLT delayed recall.
- Poisson distributions for Animal Naming.
- Multinomial distributions for PVLT delayed recognition.

These families of distributions are all computationally simple, widely used, and well understood. All are exponential families of distributions, with many favorable properties. Finally, modifications and generalizations of these families of distributions have been extensively studied, giving clear direction for model development.

Visual inspection of randomized quantile residuals^69^ was used to identify possible model misspecifications, such as overdispersion, underdispersion, and zero-inflation. In response to any misspecification identified, we chose modified and/or generalized variants of the probability distributions appropriate to address the misspecification. We considered:

- Continuous mixtures of the basic distributions, commonly used to address overdispersion, include negative binomial distributions (substituted for Poisson), beta-binomial distributions (substituted for binomial), and Dirichlet-multinomial distributions (substituted for multinomial).
- “Conway-Maxwell” generalizations of the basic distributions, including CM-Poisson^34^, CM-Binomial^37^, and CM-Multinomial^38^. These generalizations accommodate both overdispersion and underdispersion.
- Zero-inflated modifications of the above distributions.
- “N-inflated” modifications of the above distributions. These are similar to the more commonly used zero-inflated models, but rather than accommodating excess zeros, they accommodate excess of the maximum score.

Models were compared by 10-fold cross-validated log-likelihood on the unlabeled Apheleia dataset to detect evidence of overfitting. We continued this model development process iteratively until we identified a set of probability distributions that yielded no evidence of misspecification or overfitting. Once a set of probability distributions was identified, we iteratively increased the number of clusters in the model, adding additional MCI and/or dementia clusters. At each step, models were again compared by 10-fold cross-validated log-likelihood, and we inspected the fitted models for interpretability of clusters. We continued this process until the cross-validated log-likelihood decreased (indicating possible overfitting) or the clusters identified by the model were not interpretable.

Once the final model was selected, the resulting clusters (Figure 1) were examined, and interpretations of clusters (Table 2) were produced by expert discussion and consensus.

Two baseline comparator models were trained, using only patient age: a 3-cluster model, with CU, MCI, and probable dementia clusters, and a 6-cluster model, using the same structure as the final model. In both, a lognormal distribution was used to model age, and the same semi-supervised approach was used for estimation.

### Congruent Validity

As a test of congruent validity, we examined several variables in the Apheleia dataset that were not part of the clustering model, including demographics and other cognitive assessments, and which were expected to show significant between-cluster differences. All hypotheses address a classification into 3 clusters, with all MCI clusters (dMCI, mxMCI, aMCI) collapsed into a single MCI cluster, and all probable dementia clusters (maDem, saDem) collapsed into a single Dem cluster. Details of chosen variables and hypotheses are given in the Results section.

Between-cluster differences were tested with a generalized Kruskal-Wallis test.^44^ Unlike a standard Kruskal-Wallis test, this method accounts for uncertainty in cluster assignment and has greater power to detect differences when used with a probabilistic cluster assignment as produced by our model. A significant result was followed by a post-hoc pairwise comparison between all clusters, using a corresponding generalization of Dunn’s test.^70^ For both the Kruskal-Wallis and Dunn’s tests, p-values were estimated by Monte Carlo simulation^71^, drawing 10,000 samples for each test. For Dunn’s test, a step-down max-T method was applied for multiple comparison correction within each group of pairwise tests.^72,73^

### Exploration of MCI and Probable Dementia Clusters

An exploratory analysis examined the same variables in the Apheleia dataset but compared among all six clusters. We did not state any hypotheses prior to analysis. Otherwise, this analysis followed the same methods as the tests of congruent validity.

### Predictive Validity

As a test of predictive validity, we compared the model’s cluster assignments to the gold-standard diagnosis on the held-out subset (N=75) of the Rowan dataset. As we are primarily interested in distinguishing between cognitively unimpaired, MCI, and probable dementia, the model’s output is collapsed to a 3-class classification by summing the posterior probabilities of the 3 MCI clusters to a probability of MCI, and summing the posterior probabilities of the 2 Dem clusters to a probability of dementia. We report overall accuracy, with Clopper-Pearson binomial 95% confidence intervals.

In addition to overall performance, we examine performance for 4 binary classification tasks:

- Impairment classification: differentiating CU from the combination of MCI and dementia.
- Dementia classification: differentiating CU and MCI from dementia.
- CU/MCI classification: differentiating CU from MCI, under the assumption that a patient is known not to have dementia.
- MCI/dementia classification: differentiating MCI from dementia, under the assumption that a patient is known to have impairment.

These tasks were chosen as corresponding to plausible real-world use cases. We excluded two additional binary classification tasks (differentiating MCI from CU and dementia, and differentiating CU from dementia under the assumption that a patient does not have MCI) as not corresponding to any plausible use case.

For each task, we report sensitivity, specificity, positive predictive value (PPV), and negative predictive value (NPV), with Clopper-Pearson binomial 95% confidence intervals. We report the area under the ROC curve (AUROC), with a 95% confidence interval estimated by bias-corrected and accelerated (BCa) nonparametric bootstrap^74^, with 10,000 replications.

## Supplementary Material

This is a list of supplementary files associated with this preprint. Click to download.

• ApheleiaDACClusteringSupplementary.docx

## Figures and Tables

**Table 1.** Summary statistics for the Apheleia and Rowan datasets.

	Apheleia	Rowan			
	N=1189	All (N=248)	CU (N=64)	MCI (N=114)	Dem (N=70)
**Age (years)**	70.2 (9.8)	73.4 (8.2)	70.5 (7.9)	73.0 (7.6)	76.8 (8.4)
**% Female**	65.8%	51.2%	67.2%	43.0%	50.0%
**Education (years)**	15.0 (2.7)	15.0 (3.3)	17.0 (3.0)	14.5 (3.1)	14.0 (3.1)
**MMSE (0-30)**	25.8 (2.8)	25.3 (4.1)	28.0 (2.1)	26.3 (2.7)	21.1 (4.3)
**DCTClock (0-100)**	52.4 (25.4)	56.2 (27.4)	74.8 (17.3)	57.8 (23.5)	33.0 (27.2)
**DCR Recall (0-3)**	1.5 (1.1)	1.3 (1.3)	2.5 (0.7)	1.2 (1.2)	0.2 (0.7)

**Table 2.** Summary of final six cluster model results from Apheleia training dataset. Weight represents the total sample percentage identified as belonging to a given cluster after thresholding by maximum probability. Descriptions were produced by expert consensus.

Cluster	Weight	Consensus Description
CU	26.32%	Cognitively unimpaired
dMCI	20.98%	Dysexecutive MCI
mxMCI	21.10%	Mixed dysexecutive and amnestic MCI
aMCI	2.75%	Severe amnestic MCI
maDem	15.67%	Probable dementia with moderate amnestic impairment
saDem	13.18%	Probable dementia with severe amnestic impairment

**Table 3.** Model details for each DAC-derived metric. Probability models and modifications were selected based on inspection of data types, range, and distributions and upon inspection of model residuals and performance on the Apheleia dataset.

Task	Metrics (range)	Probability Model	Modification
BDST	Total Serial Order Score (0-15)	Conway-Maxwell Binomial^37^	N-inflation
Animal Naming	# Unique (0−)	Conway-Maxwell Poisson^34^	Zero-inflation
PVLT-6 Delayed Recall	# Correct (0-6)	Conway-Maxwell Binomial^37^	Zero-inflation
PVLT-6 Delayed Recognition	# Correct (0-6) # Prototypical (0-6) # Generic (0-6)	Conway-Maxwell Multinomial^38^	None

**Table 4.** Estimated cluster means and standard deviations.

		CU	dMCI	mxMCI	aMCI	maDem	saDem
**BDST**	Serial Order Score	10.9 (3.2)	8.1 (2.6)	8.3 (3.2)	8.6 (3.5)	6.0 (2.9)	5.3 (3.4)
**Animal Naming**	Unique Animals	19.2 (5.4)	14.6 (4.5)	12.7 (3.4)	19.2 (5.0)	10.0 (5.8)	8.5 (5.0)
**PVLT Delay Recall**	Correct	4.0 (1.5)	3.6 (1.2)	1.5 (1.2)	0.7 (1.4)	1.6 (1.2)	0.3 (0.6)
**PVLT Recognition**	Correct	5.9 (0.4)	5.8 (0.4)	5.0 (0.9)	3.5 (1.3)	5.2 (0.8)	2.7 (1.3)
	Prototypical Foil	0.1 (0.3)	0.2 (0.4)	0.6 (0.8)	1.6 (1.2)	0.7 (0.8)	2.2 (1.3)
	Generic Foil	0.0 (0.2)	0.0 (0.2)	0.4 (0.6)	0.9 (0.9)	0.1 (0.3)	1.1 (1.0)

**Table 5.** Between-cluster comparisons on the Apheleia dataset. Cluster sizes, means, and standard deviations are weighted by posterior probability of cluster membership. Significance is by generalized Kruskal-Wallis tests^44^, with post hoc comparisons by generalized Dunn’s tests.

	CU (N=312.1)	MCI (N=534.3)	Dem (N=341.6)	Significance and Post-Hoc Tests	
**Age (years)**	67.2 (8.8)	70.2 (9.6)	72.8 (10.0)	H=81.0 p<0.001	MCI>CU (p<0.001) Dem>CU (p<0.001) Dem>MCI (p<0.001)
**Education (years)**	15.7 (2.4)	15.0 (2.6)	14.2 (3.0)	H=70.7 p<0.001	CU>MCI (p<0.001) CU>Dem (p<0.001) MCI>Dem (p<0.001)
**MMSE (0-30)**	27.4 (1.8)	26.1 (2.3)	23.9 (3.2)	H=377.9 p<0.001	CU>MCI (p<0.001) CU>Dem (p<0.001) MCI>Dem (p<0.001)
**DCTClock (0-100)**	64.6 (21.7)	53.2 (24.0)	40.1 (25.0)	H=255.3 p<0.001	CU>MCI (p<0.001) CU>Dem (p<0.001) MCI>Dem (p<0.001)
**DCR Recall (0-3)**	2.1 (0.9)	1.5 (1.0)	1.0 (1.0)	H=271.6 p<0.001	CU>MCI (p<0.001) CU>Dem (p<0.001) MCI>Dem (p<0.001)
**PHQ-4 Depression (0-6)**	1.1 (1.4)	1.4 (1.6)	1.6 (1.7)	H=26.6 p<0.001	MCI>CU (p<0.001) Dem>CU (p<0.001) Dem>MCI (p=0.017)
**PHQ-4 Anxiety (0-6)**	1.3 (1.4)	1.5 (1.6)	1.7 (1.7)	H=12.0 p=0.002	MCI>CU (p=0.020) Dem>CU (p=0.002) Dem>MCI (p=0.036)
**NPI Apathy (0-6)**	1.0 (1.4)	1.3 (1.5)	1.3 (1.5)	H=14.7 p<0.001	MCI>CU (p=0.001) Dem>CU (p=0.004)

**Table 5.** Confusion matrix summarizing the agreement between the DAC clusters and ground-truth clinician diagnosis.

		Cluster		
		CU	MCI	Dem
**Diagnosis**	**CU**	21	0	0
	**MCI**	6	24	5
	**Dem**	0	5	14

**Table 6.** Classification performance of the model on the held-out subset of the Rowan data.

	Sensitivity	Specificity	PPV	NPV	AUROC
**Impairment**	0.907 [0.797, 0.969]	1.000 [0.839, 1.000]	1.000 [0.927, 1.000]	0.808 [0.606, 0.934]	0.985 [0.930, 0.999]
**Dementia**	0.737 [0.488, 0.909]	0.911 [0.804, 0.970]	0.737 [0.488, 0.909]	0.911 [0.804, 0.970]	0.932 [0.841, 0.975]
**CU vs. MCI**	0.829 [0.664, 0.934]	1.000 [0.839, 1.000]	1.000 [0.881, 1.000]	0.778 [0.577, 0.914]	0.977 [0.891, 0.999]
**MCI vs. Dem**	0.737 [0.488, 0.909]	0.857 [0.697, 0.952]	0.737 [0.488, 0.909]	0.857 [0.697, 0.952]	0.892 [0.750, 0.958]

**Table 7.** Comparison of predictive performance (on the held-out Rowan data) to baseline age-only models.

	DAC Model	Age-only (3 cluster)	Age-only (6 cluster)
**Overall Accuracy**	78.7% [67.7%, 87.3%]	36.0% [25.2%, 47.9%]	37.3% [26.4%, 49.3%]
**AUROC (Impairment)**	0.985 [0.930, 0.999]	0.661 [0.527, 0.777]	0.629 [0.499, 0.751]
**AUROC (Dementia)**	0.932 [0.841, 0.975]	0.664 [0.507, 0.797]	0.584 [0.404, 0.745]
**AUROC (CU vs. MCI)**	0.977 [0.891, 0.999]	0.614 [0.457, 0.749]	0.532 [0.369, 0.683]
**AUROC (MCI vs. Dem)**	0.892 [0.750, 0.958]	0.613 [0.446, 0.762]	0.532 [0.350, 0.699]

**Table 8.** Comparison of training and testing subsets of the Rowan dataset. Results in the “Significance” column are from Mann-Whitney U tests or chi-square tests of association, as appropriate.

	Train	Test	Significance
**Age (years)**	73.4 (8.1)	73.3 (8.4)	U=6711; p=0.56
**% Female**	54.3%	44.0%	*χ*^2^(1)=1.96; p=0.16
**Education (years)**	14.7 (3.3)	15.7 (3.3)	U=5599; p=0.08
**MMSE (0-30)**	25.1 (3.9)	25.7 (4.6)	U=5387; p=0.09
**DCTClock (0-100)**	54.8 (27.8)	59.3 (26.6)	U=1509; p=0.39
**DCR Recall (0-3)**	1.23 (1.26)	1.55 (1.31)	U=1445; p=0.20
**% CU**	24.9%	28.0%	*χ*^2^(2)=0.53; p=0.77
**% MCI**	45.7%	46.7%	
**% Dem**	29.5%	25.3%	
